# A Three-Dimensional (3D) Evaluation Unveiling if the Anterior Mandible Is Truly a Safe Zone for Implant Placement

**DOI:** 10.7759/cureus.38084

**Published:** 2023-04-24

**Authors:** Rutvi Vyas, Aditya Tadinada

**Affiliations:** 1 Oral and Maxillofacial Radiology, University of Florida Health, Gainesville, USA; 2 Oral and Maxillofacial Radiology, University of Connecticut, Farmington, USA

**Keywords:** sublingual hematoma, dental implants, sublingual artery, anterior mandible, cone-beam computed tomography (cbct), clinical dentistry

## Abstract

Background

Dental implants are increasingly being used in the rehabilitation of the edentulous areas in the maxilla and mandible. The anterior mandible is considered a safe zone for implant placement, but clinicians often find it challenging to control bleeding in this area. This is due to the presence of the sublingual artery, which can be of varying dimensions and can cause severe bleeding. This can be of higher significance in patients with high or uncontrolled blood pressure and in patients on blood thinners like Aspirin or Coumadin where establishing a clot can be difficult. With newer guidelines recommending that medication be discontinued only a few hours before surgery and that bleeding be managed locally, this issue has become even more challenging. With three-dimensional (3D) imaging using cone beam computed tomography (CBCT) becoming more common for implant planning, the presence of the sublingual artery can be evaluated and incorporated into the treatment plan.

The objective of this study is to evaluate the 3D location of the sublingual artery in the edentulous anterior mandible of CBCT scans of patients referred for dental implant therapy.

Methodology

A total of 50 de-identified CBCT scans with an edentulous anterior mandible referred for dental implant therapy were evaluated for this study. Cross-sectional images were generated using a CBCT reconstruction program INVIVO-5 (Anatomage, San Jose, CA, USA). After the sublingual artery was localized, measurement was conducted from a standardized point on the alveolar crest to the artery’s entry point on the lingual aspect. Measurements were also obtained from the terminal point of the artery’s course to the buccal cortical plate. Alveolar crest can either resorb or be subjected to alveoloplasty during implant placement, similar measurements were also done from a standardized point on the inferior cortical border of the mandible to the artery’s entry points on the lingual aspect. Two oral and maxillofacial radiologists conducted all measurements.

Results

It was found that the median value of the sublingual artery from the alveolar crest to the level of entry (V1) was 6.78, the vertical measurement of the artery coursing into the alveolar bone was ~4.03 mm (V2), the vertical measurement of the artery’s position within the alveolar bone at the terminal point form the crest was ~11.71 (V3), and the inferior vertical measurement from the course of the artery to the inferior border of the mandible was 9.60 mm. The artery extended about ~8.3 mm within the alveolar bone from the lingual cortex (H1), and the artery was located about 4.97 mm away from the buccal cortex (H2). Cronbach’s Alpha test showed high interoperator reliability.

Conclusions

In this retrospective study, the sublingual artery was noted to be at a critical location in the potential implant site. A site-specific evaluation using CBCT can help in localizing and avoiding perforation of the sublingual artery.

## Introduction

Dental implants are increasingly being used in the rehabilitation of the edentulous areas in the maxilla and mandible. It is very important to understand the anatomy of the area where the implants are being planned. The neurovascular supply of the area needs to be carefully analyzed before the implant is planned and placed. Understanding the complications that could potentially arise following the injury to the neurovascular bundles is critical. Uncontrolled bleeding is one such complication that could arise if the vascular supply in the area is severed accidentally [1,2]. This complication could potentially be fatal if the management of the situation has not been adequately planned. The anterior mandible is generally believed to be a safe location for dental implants [3]. However, bleeding complications in the anterior mandible, especially the floor of the mouth, could be fatal if not anticipated in advance and not appropriately planned and managed [2]. Injury to the vascular supply near the floor of the mouth can cause sublingual hematoma and could substantially cause airway obstruction [4,5]. Clinicians often find it challenging to control bleeding in this area; this is due to the presence of the sublingual artery, which can be of varying dimensions and can cause severe bleeding. This can be of greater significance in patients with high or uncontrolled blood pressure, especially with patients on blood thinners like Aspirin or Coumadin, where establishing a clot can be difficult [6]. With newer guidelines recommending that medication be discontinued only a few hours before the surgery and that bleeding be managed locally, this issue has become even more challenging [7]. With three-dimensional (3D) imaging using cone beam computed tomography (CBCT) becoming more common for implant planning, the presence of the sublingual artery can be evaluated and incorporated into the treatment plan.

The objective of this study is to evaluate the 3D location of the sublingual artery in the edentulous anterior mandible in patients referred for dental implant therapy. The study focuses on locating and tracing the path of the sublingual artery, justifying the planning of implants in the anterior mandible, and highlighting the significance of the 3D imaging modality in planning and placing the dental implants in the anterior mandible.

## Materials and methods

Anatomy of the vascular supply

**Figure 1:**
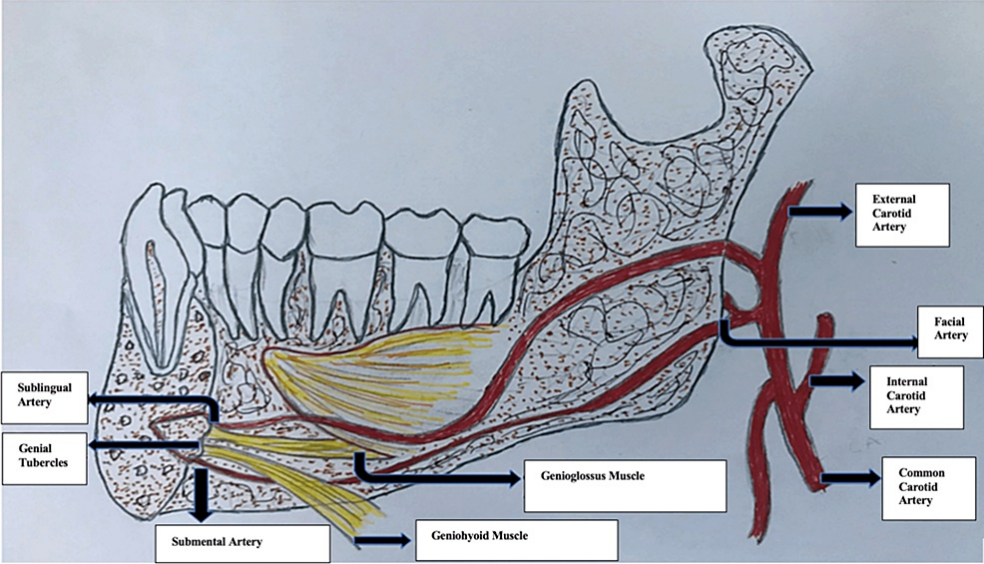
Diagrammatic representation of the vascular supply in the mandibular anterior region. Figure credits: Rutvi Vyas.

The vascular supply of the mandible and nearby anatomical structures comprises the branches of the external carotid artery, mainly the inferior alveolar artery, which is the branch of the maxillary artery, as depicted in Figure 1. The artery runs along the mandibular canal and supplies the mandible, tooth sockets, and teeth; the artery further divides into the incisive and mental branches near the premolar. The incisive branch of the inferior alveolar artery supplies the incisors to the midline where it joins the same artery on the opposite side; the facial artery, the branches of which supply the submandibular gland, lips, chin, muscles of the face, tongue, soft palate, and tonsil; and the lingual artery, which supplies the tongue and floor of the mouth. Before it terminates into the deep lingual artery at the tip of the tongue, it gives off some branches, dorsal lingual branches supplying the dorsum of the tongue and the sublingual artery supplying the sublingual gland.

**Figure 2:**
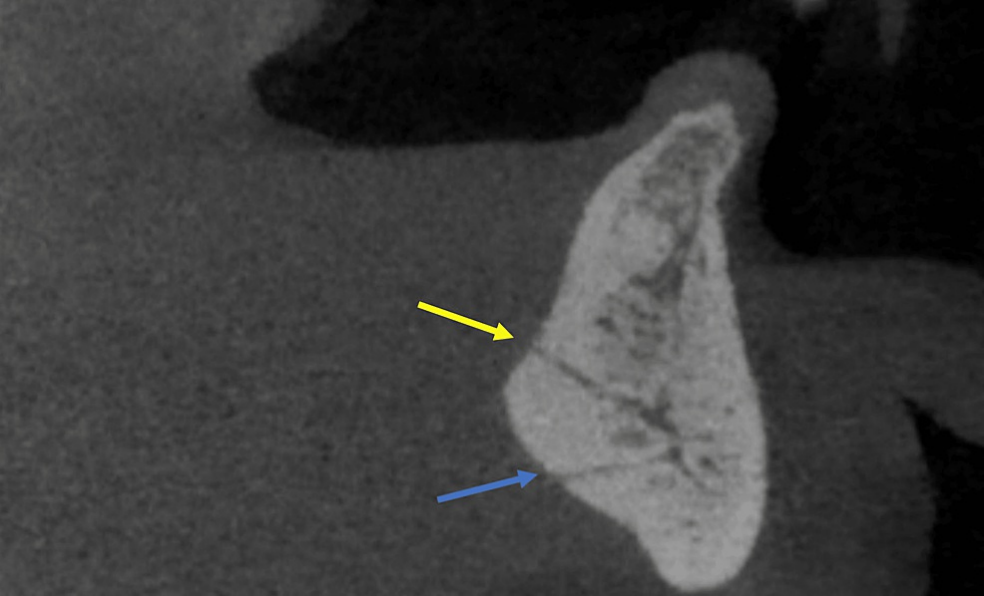
Sagittal cross-section of the CBCT scan showing superior and inferior levels of the lingual artery entering and exiting the lingual cortical plate. CBCT, cone beam computed tomography

**Figure 3:**
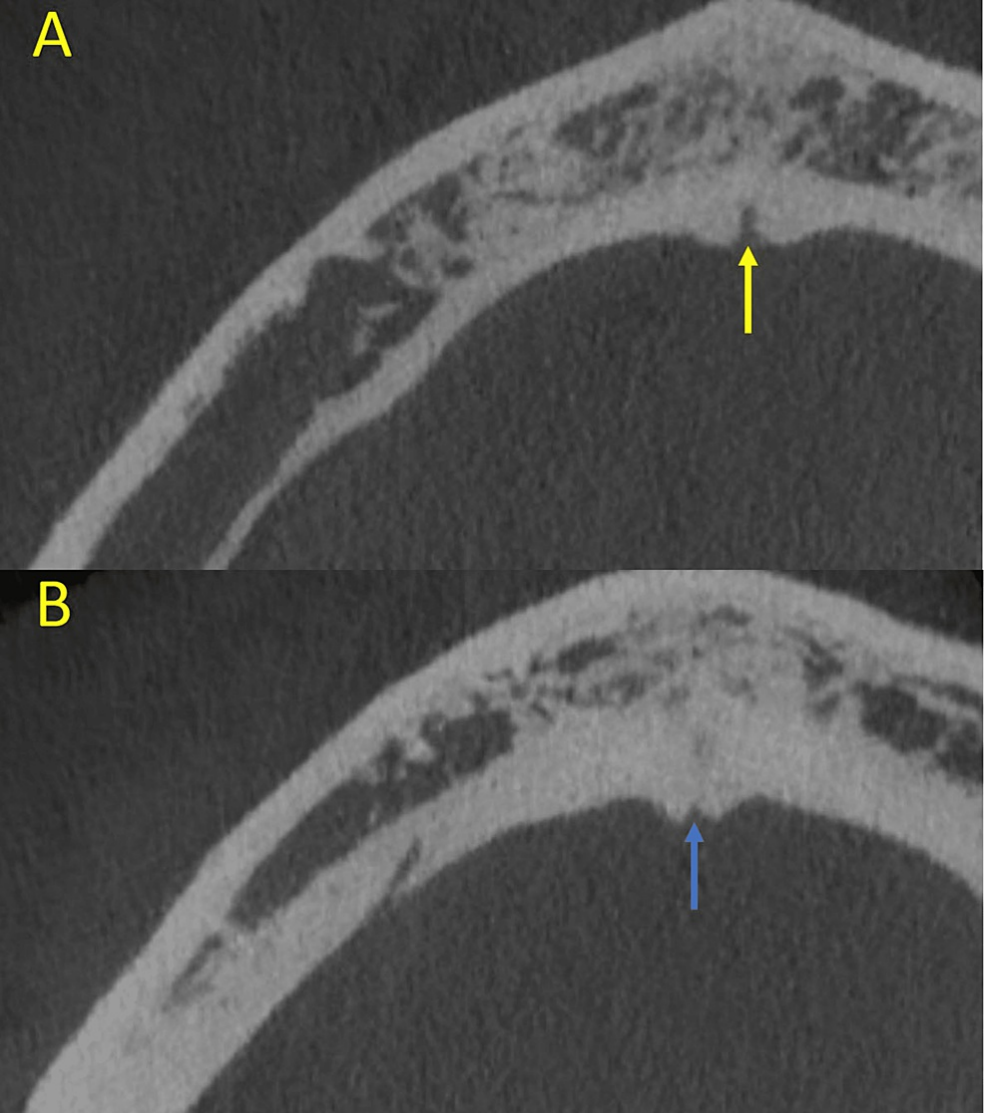
Axial cross-sections of the CBCT scan showing (A) superior level and (B) inferior level of the lingual foramen. CBCT, cone beam computed tomography

A total of 50 de-identified CBCT scans with an edentulous mandible referred for implant therapy were evaluated in the study. Cross-sectional images were generated using a CBCT reconstruction program INVIVO-5 (Anatomage, San Jose, CA, USA). The artery entering the genial tubercle on the lingual surface of the anterior mandible was traced (Figures 2, 3A, 3B). Then the course of the artery was measured in reference to the surrounding bone for implant planning to safeguard the distance from the artery.

**Figure 4:**
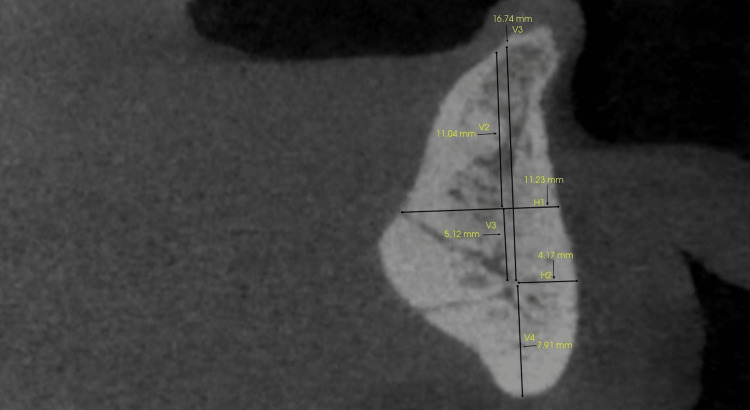
Diagrammatic representation of all measurements.

A total of six different vertical and horizontal measurements were made in reference to the artery (Figure 4). A first vertical measurement (V1) was done from a standard point (approx. 2 mm from the crest) on the crest of the alveolar ridge to the level at which the artery entered the lingual surface of the mandible. The second vertical measurement (V2) was done from the level at which the artery entered the lingual surface of the mandible to the level at the terminal point of the artery’s course from the buccal cortical plate. The third vertical measurement (V3) is from the crest to the terminal point of the artery’s course. And the fourth vertical measurement (V4) was from the inferior cortical border of the mandible to the artery’s terminal point (because the alveolar crest can either resorb or be subjected to alveoloplasty during implant placement and the values can change with time). The horizontal measurement (H1) was measured from the artery’s entry point on the lingual aspect to the buccal cortical plate, and the second horizontal measurement (H2) was measured from the terminal point of the artery’s course to the buccal plate was done. Two oral and maxillofacial radiologists conducted all measurements. All measurements were done twice with an interval of more than two weeks between sessions. Cronbach’s alpha test was done for interobserver reliability.

## Results

The mean and median values of all the measurements were obtained (Table 1). It was found that the median value of the sublingual artery from the alveolar crest to the level of entry (V1) was 6.78, the vertical measurement of the artery coursing into the alveolar bone was ~4.03 mm (V2), the vertical measurement of the artery’s position within the alveolar bone at the terminal point from the crest was ~11.71 (V3), and the inferior vertical measurement from the course of the artery to the inferior border of the mandible was 9.60 mm. The artery extended about ~8.3 mm within the alveolar bone from the lingual cortex (H1), and the artery was located about 4.97 mm away from the buccal cortex (H2). The overall range of the measurements inclusive of both the observer’s findings were as follows: H1, 3.89-13.8 mm; H2, 2.36-9.02 mm; V1, 1.17-22.28 mm; V2, 1.63-6.99 mm; V3, 2.74-25.21 mm; and V4, 5.25-13.28 mm. The interobserver reliability with Cronbach's alpha was highly significant with the *P*-value < 0.001 (Table 2).

**Table 1:** Descriptive analysis. SD, standard deviation; CI, confidence interval

Measurement	Observer 1					Observer 2				
	Mean	±SD	Range	Median	95% CI	Mean	±SD	Range	Median	95% CI
H1	8.714	2.349	4.06-13.80	8.84	7.03-10.43	8.875	2.444	3.89-12.89	9.18	7.09-11.17
H2	5.105	1.651	2.36-8.82	4.89	3.98-6.14	5.156	1.679	2.41-9.02	5.05	3.86-6.12
V1	7.017	4.297	1.23-22.28	6.78	3.53-9.71	7.035	4.303	1.17-22.21	6.70	3.42-9.76
V2	4.089	1.457	1.75-6.97	4.03	2.78-5.02	4.132	1.453	1.63-6.99	4.02	2.89-5.16
V3	11.310	5.040	2.74-25.06	11.66	6.73-15.78	11.350	5.031	2.76-25.21	11.71	6.74-15.79
V4	9.364	2.168	5.25-13.28	9.60	7.26-10.86	9.351	2.154	5.31-12.87	9.55	7.27-11.12

**Table 2:** Interobserver reliability. NS: *P* > 0.05 (not significant). ^*^P < 0.05 (significant). ^**^P < 0.001 (highly significant). SEM, standard error of mean; CI, confidence interval; $Paired t-test, #Pearson correlation coefficient

Measurement	Cronbach’s alpha	95% CI		*P*-value	*r*-value^#^	Difference in Obs1 and Obs2^$^		
		Lower	Upper			Mean	SEM	*P-*value
Overall	0.999	0.998	0.999	<0.001**	0.998	0.0498	0.0201	0.014*
H1	0.985	0.968	0.993	<0.001**	0.971	0.1610	0.1076	0.145; NS
H2	0.999	0.997	0.999	<0.001**	0.997	0.0510	0.0233	0.037*
V1	1.000	0.999	1.000	<0.001**	1.000	0.0180	0.0248	0.473; NS
V2	0.998	0.996	0.999	<0.001**	0.997	0.0430	0.0219	0.060; NS
V3	1.000	1.000	1.000	<0.001**	1.000	0.0397	0.0204	0.062; NS
V4	0.999	0.998	0.999	<0.001**	0.998	0.0137	0.0261	0.605; NS

## Discussion

In our study, we localized the position of the sublingual foramen from the nearby alveolar bone limits. It is important to evaluate the location of the lingual foramina in the anterior mandible to avoid any injury to the vasculature traversing through the foramina. The position of this foramina may vary based on the amount of alveolar bone resorption in the area. Studies have shown variation in the position of the sublingual foramen based on the age of the patient as well as based on gender [8]. Violation of the site can lead to a life-threatening situation. Severe bleeding in the area can obstruct the airway and can potentially be life-threatening if not managed appropriately.

3D evaluation of the area has become pivotal in the success of implant planning. It provides an accurate evaluation of the bone volume in the area along with the proximity to the nearby anatomical structures. The bone values obtained using CBCT have high accuracy almost comparable to the actual measurement obtained directly from measuring the skull bone thickness [9].

In our study, we observed two foramina along the lingual surface of the anterior mandible and recognized them as superior and inferior levels of the sublingual artery/lingual foramen. The different vertical and horizontal measurements were obtained in reference to these entry points of the sublingual foramen. We also observed that these two entry foramina extend along the alveolar bone and anastomose somewhere along the anterior third of the alveolar bone width. Hence, we measured the two horizontal measurements that are H1, which provided the entire thickness of the alveolar bone from the superior entry point of the sublingual artery to the labial cortical surface, and H2, which provided the available labial bone thickness at the junction of the anastomosis of the two branches. These measurements are critical, especially if any bone augmentation is planned along the labial cortical surface [10]. 

We also included vertical measurements in our study. The purpose of the superior vertical measurements above the sublingual artery to the crest was to determine the available bone height. The alveolar bone is prone to resorption following the extraction in the area. Considering the inclined course of the artery in the alveolar bone, the vertical height was measured at the entry of the artery in the bone as well as at the anastomosis level. The course of the artery helps determine the position of the implant in the labiolingual direction based on the proximity to the vasculature. We also considered the fourth vertical measurement in our study that is V4, which measures the inferior height of the alveolar bone from the sublingual artery. The significance of this measurement is to consider the bone integrity in the area, especially in cases of severe bone resorption to determine the future strength of the jaw. Bone resorption in the jaw starts at the crestal level, and hence, the bone height at the inferior third of the mandible generally remains steady following crestal ridge resorption.

Literature reports several fatal hemorrhages followed by floor-of-the-mouth hematoma due to vascular perforation during the implant placement, even penetrating through the lingual cortical surface and injuring the sublingual artery, leading to life-threatening conditions [11-13]. Several treatment options were mentioned in the literature, starting with achieving hemostasis with compression, hemostatic agents, and vasculature ligation, as well as the opening of the airway with endotracheal intubation, tracheostomy, and other procedures to stop the bleeding and securing the airway [14,15].

This study confirms that variation in the bone height and subsequently the location of the sublingual artery add risk to implant placement if not carefully planned. It is particularly important in the edentulous ridges, which are more prone to resorption and eventually loss of bone volume. The combination of bone loss with proximity to the critical anatomical structure needs to be carefully evaluated. Preplanning the area with the 3D imaging modality can help avoid such mishaps. In our study, we observed that the different measurements obtained for the potential implant site varied in range. When evaluated in two-dimensional (2D) images such as a panoramic radiograph, one needs to understand the principles of image formation before determining the available bone height or even the proximity to the anatomical structures. In a panoramic radiograph, due to the caudocranial nature of the X-ray beam, the structures that are closer to the X-ray beam that is lingually positioned are projected higher up in the radiograph as compared to the buccal objects [16]. So, in the case of the sublingual artery, its course can be varied as it penetrates through the alveolus in the labiolingual direction and it would be difficult to determine its location in a 2D radiograph just by tracing the genial tubercle, which is a radiopaque structure housing the lingual foramen. The assumption made with 2-D imaging can be misleading and can lead to life-threatening situations if not handled appropriately, and hence, the clinician must preplan the implant site thoroughly with 3D imaging such as CBCT.

The limitations of the study included that our study sample only included edentulous mandibular scans, which significantly reduce artifacts such as scatter and beam hardening in the scan, which are generally associated with metallic restorations in dentate patients. The resolution of the scan plays a significant role in evaluating and measuring the fine details within the CBCT scan. The other limitation of the study included our relatively small sample size, and a study with a larger sample size will help in further understanding the concept.

## Conclusions

Although the anterior mandible is very favorable for implant placement, the area in the midline in proximity to the lingual foramen needs to be carefully evaluated. 3D imaging modality plays a critical role in the analysis of the overall bone quality, quantity, and proximity to the nearby anatomy.
